# Case Report: Clinical manifestations reported in a canine after ingesting ruxolitinib phosphate (Opzelura^®^)

**DOI:** 10.3389/fvets.2026.1757633

**Published:** 2026-02-12

**Authors:** Leah D. Swanson, Renee D. Schmid, Steven Butt, Lynn R. Hovda

**Affiliations:** 1 SafetyCall International, Bloomington, MN, United States; 2 Pet Poison Helpline, Bloomington, MN, United States; 3 BluePearl Pet Hospital, Phoenix, AZ, United States

**Keywords:** apoquel^®^, JAK inhibitors, oclacitinib maleate, Opzelura^®^, ruxolitinib phosphate

## Abstract

This case report describes a canine patient that developed cardiovascular and ocular abnormalities following ingestion of ruxolitinib phosphate 1.5% topical cream. The objective of this report is to characterize the exposure history, clinical presentation, diagnostic findings, therapeutic interventions, and to evaluate ruxolitinib phosphate within the context of its pharmacologic class, emphasizing similarities with related veterinary medications. Building on a recently published retrospective study of oclacitinib maleate overdoses, this case highlights shared clinical manifestations of ruxolitinib phosphate overdose and contributes to a broader understanding of JAK inhibitor toxicosis in veterinary medicine.

## Introduction

Janus kinase (JAK) inhibitors are a class of immunomodulatory agents frequently prescribed in human and veterinary medicine to suppress pro-inflammatory cytokines and interleukins (IL). These medications selectively inhibit a subgroup of protein tyrosine kinases, JAK1, JAK2, JAK3, and tyrosine kinase (TYK) 2, which serve as mediators in cytokine signaling that regulates inflammation and immune response (1, 2). Among the JAK family, JAK1 plays a critical role mediating several cytokines and interleukins, including IL-2, IL-4, IL-6, IL-13, and IL-31, which are primarily associated with inflammation and pruritis (3). In contrast, the hematopoietic signaling pathways are primarily affected by JAK2 and JAK3 pathways. Myeloid and erythroid cell lines are largely associated with JAK2, whereas JAK3 is involved with lymphoid cell development. Immune defense mediated by type 1 interferons, as well as IL-12 and IL-23, is regulated through TYK2 signaling (3).

Ruxolitinib phosphate (Jakafi^®^) was the first drug in the JAK inhibitor class to receive approval from the U.S. Food and Drug Administration (FDA) in 2011 for the management of myelofibrosis in humans (4). A topical cream formulation of ruxolitinib phosphate (Opzelura^®^) was subsequently approved in 2021 for the treatment of atopic dermatitis and nonsegmental vitiligo (1). The topical formulation of ruxolitinib phosphate is labeled for external application twice daily to affected areas, with use restricted up to 20% of body surface area and a maximum of one 60-g tube per week (1). Two JAK inhibitors in veterinary medicine, oclacitinib maleate (Apoquel^®^) and ilunocitinib (Zenrelia^®^), are FDA approved for use in canines for the treatment of atopic and allergic dermatitis. A recently published retrospective study evaluating oclacitinib maleate toxicosis in veterinary patients, as reported to Pet Poison Helpline^®^ (PPH), identified a spectrum of clinical effects including neurological, gastrointestinal, ocular, cardiovascular, hematological, and multi-organ injury (5). Additional reports, including a brief communications article (6) and a separate case report involving a canine treated for oclacitinib maleate toxicosis, (7) also described cardiovascular and ocular abnormalities. The clinical signs observed in the present case reflect these shared findings and may be attributed to common pharmacodynamic properties of JAK inhibitors such as oclacitinib maleate and ruxolitinib phosphate.

As a selective JAK1 and JAK2 inhibitor, ruxolitinib phosphate exhibits rapid absorption, wide volume of distribution, and significant hepatic metabolism via cytochrome P450 enzyme CYP3A4 following oral administration (8, 9). Peak plasma concentrations are achieved within 2 h after ingestion with 50% bioavailability in canines (9). Ruxolitinib phosphate is primarily eliminated as multiple metabolites with 34–36% in urine and 55–58% in feces, with negligible excretion (< 1%) of the parent compound, reflecting ruxolitinib phosphate's extensive metabolism prior to elimination in canines (10).

Despite the popularity of JAK inhibitors in veterinary and human medicine, toxicological data on the effects of overdose in veterinary patients remains poorly understood. A recent retrospective study examining oclacitinib maleate toxicosis proposed that the resulting effects could be mediated through mechanisms such as a rebound cytokine dysregulation syndrome or mast cell degranulation (5). Similar observations are described in human medicine, where abrupt discontinuation of JAK inhibitors without tapering has been linked to rebound hyperinflammatory syndromes (11). A human case series of 47 patients with myelofibrosis who abruptly discontinued ruxolitinib phosphate due to loss of clinical benefit and/or drug-related adverse effects found that 11% developed signs of withdrawal consistent with cytokine rebound syndrome, including splenomegaly, anemia, thrombocytopenia, disseminated intravascular coagulation, and effusions (11). Gradual tapering of ruxolitinib phosphate and other JAK inhibitors has been recommended in human medicine to reduce the risk of adverse effects, with consideration given to initiating corticosteroids along with other supportive therapies (11). The authors hypothesize that JAK inhibitor toxicosis in canines may closely resemble the rebound cytokine syndrome observed following cessation of JAK inhibitor therapy in humans.

Although JAK inhibitors are intended to exert anti-inflammatory effects due to cytokine suppression, they may paradoxically amplify macrophage driven inflammation in response to toll-like receptor 4 (TLR4) agonists which trigger cytokine release (12, 13). These TLR4s can initiate inflammatory cascades involving nuclear factor-kappa B (NF-kB), tumor necrosis factor- α (TNF- α), IL-6, and IL-1β, potentially leading to paradoxical hyperinflammation (13). As understanding of JAK inhibition in immune regulation has evolved, it has become evident that different JAK inhibitors may exert variable immunologic effects depending on their receptor selectivity. While ruxolitinib phosphate is classified as a JAK1/JAK2 inhibitor, evidence suggests that many of its immunomodulatory effects are predominantly JAK1 mediated, similar to findings reported in oclacitinib maleate overdose. In a study by Schönberg et al., ruxolitinib impaired NK cell maturation, potentially increasing infection risk, whereas the selective JAK2 inhibitor pacritinib caused significantly less NK cell dysfunction, supporting a stronger JAK1 role, especially in withdrawal syndromes (14). This JAK1 preference may therefore help explain the overlapping clinical manifestations observed in overdoses of both ruxolitinib phosphate and oclacitinib maleate (15).

Highly vascularized organs, such as the eyes, are particularly sensitive to inflammation during systemic immune responses. Consequently, veterinary patients experiencing JAK inhibitor toxicosis often present with ocular abnormalities within a few hours of exposure based on cases reported to PPH. In this specific case of ruxolitinib phosphate exposure and other cases which involve oclacitinib maleate, common ocular signs include periocular edema, third eyelid elevation, ptosis, and less frequently, keratoconjunctivitis sicca (KCS), glaucoma, and uveitis. A study evaluating the role of cytokines in ocular diseases reported elevated levels of pro-inflammatory cytokines in individuals with excessive immune reactivity, such as those with allergies (16). Furthermore, a study by Leonardo demonstrated elevated levels of specific interleukins in the tears of allergic individuals, including IL-1β, IL-2, IL-5, IL-6, IL-12, IL-13, and monocyte chemoattractant protein-1 (MCP-1) (17). Moreover, elevated IL-6 levels have been linked to various other inflammatory conditions including glaucoma, uveitis, KCS and allergic eye diseases (18). In early phases of toxicosis with JAK inhibitors, profound suppression of cytokine signaling disrupts normal autonomic control of ocular structures, impairing sympathetic tone to the eyelids and lacrimal glands (18). This autonomic imbalance, coupled with endothelial leakage and vascular instability, may also explain the development of these ocular signs.

Ruxolitinib phosphate, among other JAK inhibitors, has been associated with cardiovascular effects in both human and veterinary medicine. In 2021, the FDA issued a black box warning for all JAK inhibitors, including both oral and topical forms of ruxolitinib phosphate, citing a potential risk of sudden death in patients with pre-existing cardiovascular disease (19). This warning may be partly explained by the cardiovascular effects of systemic inflammation. In a hyperinflammatory state, IL-6 has been implicated in affecting the sinoatrial node, increasing vagal tone, and contributing to bradycardia (20). Additionally, this effect could be compounded by β2 adrenergic-receptor dysregulation from pro-inflammatory cytokines which reduces responsiveness to catecholamines (21). Activation of β2-adrenergic receptors by cytokines induces vasodilation, leading to reduction in vascular resistance and a compensatory reflex tachycardia to maintain blood pressure. However, with prolonged stimulation, these receptors become desensitized and downregulated, diminishing the heart's responsiveness to sympathetic stimulation and ultimately contributing to bradycardia (21). *In vivo* safety studies of ruxolitinib phosphate in canines demonstrated cardiovascular effects at supratherapeutic exposures, approximately 36 to 49-fold greater than maximum therapeutic human dose (10). Reported effects included decreased systolic and diastolic blood pressure, and reduced mean and pulse arterial pressure, with a compensatory tachycardia (10).

In the experience of PPH veterinarians, acute JAK inhibitor toxicosis typically presents as a biphasic syndrome in cats and dogs. Initially, profound JAK-STAT blockade suppresses cytokine and interleukin activity, dampening inflammatory signaling, and disrupting autonomic regulation. This results in cardiovascular and ocular signs, altered mentation, transient pancytopenia, and electrolyte abnormalities such as hypokalemia and hypophosphatemia. As the drug is metabolized, cytokine signaling rebounds in a dysregulated manner, with loss of inhibitory feedback triggering targeted vascular inflammation and ischemic injury in highly vascular organs, leading to progression of cardiovascular signs, development of ocular inflammation, and the onset of acute renal injury and myositis.

The objective of this case report is to document the similarities seen with ruxolitinib phosphate toxicosis with other veterinary JAK inhibitors. With the growing use of this drug class and anticipated development of new agents within this class, prompt recognition of overdose manifestations is essential to guide timely supportive care and mitigate the risk of adverse outcomes.

## Case Report description

A 4-year-old female spayed Labrador retriever mix weighing 32.2 kg was presented to an emergency veterinary hospital approximately 6–8 h after ingestion of an estimated 45 g of a 60 g tube of 1.5% ruxolitinib phosphate ointment (Opzelura^®^), resulting in an approximated ingestion of 21 mg/kg. The owners reported lethargy and ptosis approximately 3 h post-ingestion. On presentation, the patient was bright, alert, and responsive but anxious. Physical examination findings included tachycardia with a heart rate of 200 bpm, respiratory rate of 50 breaths per min with normal effort, body temperature of 100.3°F (37.9 °C), and systolic blood pressure of 138 mmHg. Mucous membranes were pink and tacky with a capillary refill time under 2 s. Bilateral ptosis was noted. Sinus tachycardia was present on ECG; no murmurs or arrhythmias were auscultated. See Table 1 for timeline of clinical signs.

**Table 1:** Timeline of clinical signs that develops during the clinical course.

**Event**	**0 h**	**3 h**	**6–8 h**	**18–20 h**	**25 h**
**Description**	**Ingestion**	**Initial clinical signs**	**Presentation to clinic**	**Hospitalization**	**Discharge**
Clinical signs and conditions	N/A	Lethargy, ptosis OU	Anxious, sinus tachycardia, KCS	Sinus bradycardia	Discharged with persistent bradycardia

Initial diagnostics included a Schirmer Tear Test revealing markedly reduced tear production (0 mm OD, 6 mm OS). Blood submitted for analysis (see Tables 2, 3) showed mild neutropenia (2.52 *K*/ul, 2.95–11.64 *K*/ul), increased lactate (5.42 mmol/*L*, 0.6–3 mmol/*L*), and an elevated alanine aminotransferase (ALT; 129 *U*/*L*, 10–125 *U*/*L*); the remainder of the complete blood count (CBC) and chemistry panel was within normal limits. Following consultation with PPH, intravenous Lactated Ringer's Solution (3.7 ml/kg/h, 90 ml/kg/day) was administered. One dose each of butorphanol (0.3 mg/kg IV) and acepromazine (0.06 mg/kg IV) were administered for persistent tachycardia and mild anxiety. Throughout hospitalization, continuous ECG monitoring revealed sinus rhythm with variable heart rates, without evidence of arrhythmias. The patient maintained an excellent appetite. Supportive care initiated at time of hospitalization and continued until discharge included S-Adenosyl-L-Methionine (SAMe)/silybin (26.4 mg/kg PO q 24 h), omeprazole (0.62 mg/kg PO q 12 h), maropitant (1 mg/kg IV q 24 h), ophthalmic lubrication (OU q 2 h), and enteral nutrition. The patient developed a sinus bradycardia during hospitalization, with the heart rate dropping as low as 60 bpm just prior discharge (bradycardia defined in this patient was a heart rate below 100 bpm in a clinical setting). Systolic blood pressure remained normal with a range of 104 mmHg to 138 mmHg. Repeat CBC (see Table 2) revealed anemia (HCT 33.8%, 37.3–61.7%; HGB 11.6 g/dl, 13.1–20.5 g/dl; RBC 4.85 x 1012/*L*, 5.65–8.87 x 1012/*L*), thrombocytopenia (147 K/ul, 148–484 K/ul), and a decreased reticulocyte count (9.7 K/ul, 10–110.0 K/ul). Serum chemistry showed mild increase in ALT (156 *U*/*L*, 10–125 *U*/*L*) and decreased amylase (408 *U*/*L*, 500–1500 *U*/*L*). Extended hospitalization was advised due to persistent sinus bradycardia but not pursued by the owner due to cost concerns. The patient was prescribed a 14-day course of Denamarin and Genteal Artificial Tears to be administered three to four times daily pending recheck. Upon discharge, the patient was in a bright and alert state after 25 h of hospitalization, with instructions for follow-up diagnostics to be completed by the primary veterinarian. According to the owner, all clinical signs and clinicopathologic abnormalities had resolved on follow-up evaluations.

**Table 2:** Initial and follow-up clinicopathologic findings.

**Lab results:**	**6–8 h post-ingestion**	**18–20 h post-ingestion**	**Reference Interval**
**HCT**	48.9 %	**33.8 %**	37.3–61.7 %
**HGB**	16.9 g/dl	**11.6 g/dl**	13.1–20.5 g/dl
**MCHC**	34.6 g/dl	34.3 g/dl	32.0–37.9 g/dl
**WBC**	5.89 *K*/ul	8.21 *K*/ul	5.05–16.76 *K*/ul
**LYMPHS**	2.89 *K*/ul	4.06 *K*/ul	1.05–5.10 *K*/ul
**%LYMPHS**	49.1 %	49.5%	N/A
**MONOS**	0.36 *K*/ul	0.46 *K*/ul	0.16–1.12 *K*/ul
**%MONOS**	6.1 %	5.6%	N/A
**NEUT**	**2.52** ***K*****/ul**	3.59 *K*/ul	2.95–11.64 *K*/ul
**%NEUT**	42.8 %	43.7 %	N/A
**EOS**	0.12 *K*/ul	0.09 *K*/ul	0.06–1.23 *K*/ul
**%EOS**	2.0 %	1.1 %	
**BASO**	0.00 *K*/ul	0.01 *K*/ul	0.00–0.10 *K*/ul
**%BASO**	0.0 %	0.1%	
**PLT**	**147** ***K*****/ul**	**147** ***K*****/ul**	148–484 *K*/ul
**RETIC-HGB**	28.5 pg	26.6 pg	22.3–29.6
**Retics**	105.9 ***K***/ul	**9.7** ***K*****/ul**	10.0–110.0 *K*/ul
**%Retics**	1.5 %	0.2 %	N/A
**RBC**	7.06 ***M***/ul	**4.85** ***M*****/ul**	5.65–8.87 ***M***/ul
**MCV**	69.3 fL	69.7 fL	61.6–73.5 fL
**MCH**	23.9 pg	23.9 pg	21.2–25.9 pg
**RDW**	17.3 %	14.0 %	13.6–21.7 %
**MPV**	12.0 fL	12.0 fL	8.7–13.2 fL
**PDW**	11.5 fL	13.0 fL	9.1–19.4
**PCT**	0.24 %	0.18 %	0.14–0.46
**ALB**	3.2 *g*/dl	2.8 g/dl	2.3–4.0 g/dl
**ALKP**	60 *U*/*L*	45 *U*/*L*	23–212 *U*/*L*
**ALT**	129 *U*/*L*	156 *U*/*L*	10–125 *U*/*L*
**AMYL**	678 *U*/*L*	408 *U*/*L*	500–1,500 *U*/*L*
**BUN/UREA**	18 mg/dl	21 mg/dl	7–27 mg/dl
**Ca**	9.4 mg/dl	9.2 mg/dl	7.9–12.0 mg/dl
**CHOL**	222 mg/dl	186 mg/dl	110–320 mg/dl
**CREA**	1.7 mg/dl	1.5 mg/dl	0.5–1.8 mg/dl
**GGT**	7 *U*/*L*	4 *U*/*L*	0–11 *U*/*L*
**GLU**	130 mg/dl	89 mg/dl	74–143 mg/dl
**LIPA**	1,096 *U*/*L*	1,042 *U*/*L*	200–1,800 *U*/*L*
**PHOS**	2.7 mg/dl	4.3 mg/dl	2.5–6.8 mg/dl
**TBIL**	0.1 mg/dl	< 0.1 mg/dl	0.0–0.9 mg/dl
**TP**	6.2 g/dl	5.7 g/dl	5.2–8.2 g/dl
**GLOB**	3.1 g/dl	2.9 g/dl	2.5–4.5 g/dl
**ALB/GLOB**	1.0	1.0	N/A
**BUN/CREA**	10	14	N/A

Results in bold indicate abnormal value.

**Table 3:** EPOC venous blood gas 6–8 h post exposure.

**Analyte**	**Result**	**Reference range**	**Critical range**	**Reportable range**
**pH**	7.396	7.360–7.4690	5.500–9.000	6.500–8.000
**pCO2**	32.7 mmHg	30–47.0	4.0–251.0	5.0–250.0
**pO2**	**69.5 mmHg**	24.0–54.0	4.0–751.0	5.0–750.0
**Na+**	151 mmol/L	140–151	84–181	85–180
**K+**	3.7 mmol/L	3.5–5.0	0.5–13.0	1.5–12.0
**Cl-**	115 mmol/L	106–127	64–141	65–140
**Ca++**	1.38 mmol/L	1.13–1.42	0.00–5.00	0.25–4.00
**TCO2**	19.3 mmol/L	17.0–26.0	4.0–51.0	5.0–50.0
**Glu**	121 mg/dl	63–124	18–702	20–700
**Lac**	**5.42 mmol/L**	0.60–3.00	0.00–21.00	0.30–20.00
**BUN**	17 mg/dl	7–26	2–121	3–120
**Crea**	1.47 mg/dl	0.40–1.50	0.29–15.01	0.30–15.00
**HCT**	45 %	36–55	9–76	10–75
**cHgb**	15.2 g/dl	12.0–19.0	2.3–26.0	3.3–25.0
**cHCO3-**	20.1 mmol/L	16.−28.0	0.0–86.0	1.0–85.0
**BE(ecf)**	−4.8 mmol/L	−5.0–5.0	−31.0–31.0	−30.0–30.0
**BE(b)**	−3.8 mmol/L	−4.0–4.0	−31.0–31.0	−30.0–30.0
**cSO2**	93.8 %	40.0–90.0	−0.1.0–101.0	0.0–100.0
**AGapK**	21 mmol/L	5–22	−11–100	−10–99
**BUN/Crea**	11.4 mg/mg	0.2–400.0	0.1–400.1	0.2–400.0

Results in bold indicate abnormal value.

## Discussion

The canine in this case report exhibited cardiovascular and ocular manifestations comparable with oclacitinib maleate toxicosis in patients with suspected cytokine dysregulation. Tachycardia, noted at the time of initial presentation, may be attributed to the combination of mild anxiety and the exposure of ruxolitinib phosphate. Sedatives were initially administered to address tachycardia, as they are often recommended as first-line therapy due to their ability to reduce sympathetic tone, and thereby improving tachycardia, without further compromising cardiovascular stability. The clinical progression to bradycardia is consistent with the expected pathophysiological cascade of an exaggerated cytokine rebound syndrome which typically begins with tachycardia and progresses to bradycardia (6). These findings are also consistent with cardiovascular effects documented in canines exposed to high doses of ruxolitinib phosphate *in vivo* safety studies (10). Hallmark ocular changes were also observed in this patient, similar to those reported with other JAK inhibitors. As previously noted, the eye's extensive vascular supply contributes to its heightened sensitivity during systemic immune responses. In addition, autonomic dysfunction affecting eyelid position and lacrimal gland innervation may have contributed to the development of ptosis and a subsequent reduction in tear production, culminating in KCS. Given the mechanism of action associated with JAK inhibitor toxicosis and the presumed pathophysiology underlying the cause of ocular signs including inflammation and autonomic dysregulation, these findings are expected to be transient and self-limiting; however symptomatic therapy such as artificial tears and topical immunomodulatory agents to support tear production and corneal health are advised as needed.

Hematologic evaluation of the patient in this report revealed mild anemia, neutropenia, thrombocytopenia, a mild elevation in ALT, and an elevated lactate concentration. Anemia and thrombocytopenia are among the most frequently reported adverse events associated with ruxolitinib phosphate at therapeutic doses in humans (22). The slight reduction in HCT and platelet count is likely be attributable to hemodilution following intravenous fluid therapy, however in other cases of JAK inhibitor toxicosis reported to PPH, hematologic changes on CBC have been observed due to early leukocyte redistribution (within 1–12 h) or later bone marrow suppression associated with JAK2 pathway inhibition. The initial mild neutropenia may reflect a developing inflammatory response, and the mild increase in ALT, particularly when considered alongside elevated lactate, may be consistent with transient hepatic insult, early tissue hypoxia, or the broader systemic inflammatory effects associated with ruxolitinib phosphate toxicosis. In accordance with this systemic inflammatory response, elevated pro-inflammatory cytokines can exert direct effects on the liver. Notably, IL-6 is extensively recognized to trigger upregulation of C-reactive proteins (CRP) and other acute-phase proteins (23). This cytokine-driven inflammatory cascade may partly explain the mild elevation of ALT appreciated in this patient. Without evaluation of aspartate aminotransferase (AST) and/or creatine kinase (CK), however, inflammation of the muscle cannot be ruled out.

Management of clinical signs in cases of JAK inhibitor toxicosis involves symptomatic and supportive care across multiple organ systems. Intravenous fluid therapy should be guided by the patient's clinical presentation, as affected patients often exhibit gastrointestinal and cardiovascular signs that may necessitate higher initial fluid rates. The rate should be tailored according to resuscitation requirements, hydration status, and perfusion parameters, with frequent reassessment as the clinical course evolves. In cases of refractory hypotension, vasopressor support may be indicated. Additional cardiovascular stabilization may include the use of beta blockers in the normotensive tachycardic patient or atropine for the bradycardic patient. Given the potential for a hyperinflammatory response associated with dysregulated cytokine signaling, ongoing adjustment of therapy is essential, as increases in vascular permeability may contribute to endothelial instability and peripheral edema (24). Anecdotally, intravenous lipid emulsion has been used successfully to provide cardiovascular support and treatment in veterinary patients with severe hypotension and tachycardia. In cases of refractory cardiovascular signs, additional diagnostic evaluation may be warranted, including measurement of serum cardiac troponin concentrations, echocardiography, and/or consultation with a veterinary cardiologist. If severe cardiovascular signs persist without treatment, evidence of myocardial involvement may develop, including troponin elevation and echocardiographic changes. Ocular signs should be treated symptomatically with ophthalmic medications with referral for specialized care with an ophthalmologist in cases with prolonged or severe ocular sequela. Per PPH data, hematologic changes may be seen within 72 h, therefore, laboratory monitoring is recommended for a minimum of 72 h and extended as clinically indicated. Given the pharmacokinetic profile of ruxolitinib phosphate, intravenous lipid emulsion and hemodialysis may be considered when available, recognizing that no published veterinary reports currently prove efficacy for their use with JAK inhibitor toxicosis. In the human literature, a case of ruxolitinib discontinuation syndrome complicated by severe respiratory failure and acute respiratory distress syndrome (ARDS) describes the use of extracorporeal life support (ECMO) and corticosteroid therapy, with reported improvement in respiratory status following initiation of these supportive interventions (25).

In this case, hospitalization until resolution of signs was not pursued due to financial constraints. As is frequently encountered in veterinary medicine, financial limitations influenced the extent of diagnostic and therapeutic interventions pursued. Consequently, more intensive in-hospital monitoring and additional diagnostic assessment that may have provided further clinical insight were not performed. Despite the potentially life-threatening nature of JAK inhibitor toxicosis at lower dosages as reported to PPH, the patient in this case made a full recovery and survived per follow-up information provided from the owner, however a specific timeframe of resolution of signs was not available.

## Conclusion

This case report illustrates the importance of recognizing potential clinical implications associated with overdose of JAK inhibitors in veterinary patients, particularly given the overlap in clinical signs observed within this pharmacologic class. Moreover, it underscores the necessity of disseminating this information to prescribing doctors and pet owners to promote safe storage and handling, thereby minimizing the likelihood of accidental overdose exposures. As their use continues to expand in both human and veterinary medicine, JAK inhibitors are expected to remain a mainstay in therapeutic practice. Given their expanding prevalence, veterinary professionals must stay vigilant about the safe use of these drugs and continue educating clients about their potential hazards, ensuring both optimal treatment outcomes and patient safety. Equally important is consistent follow-up, both in clinical management and in documentation of similar cases, as it allows the veterinary community to refine its understanding, improve safety guidelines, and continue making meaningful progress in preventing and managing JAK inhibitor related toxicities.

## Data Availability

The raw data supporting the conclusions of this article will be made available by the authors, without undue reservation.
